# Integrating Artificial Intelligence and Wearable IoT System in Long-Term Care Environments

**DOI:** 10.3390/s23135913

**Published:** 2023-06-26

**Authors:** Wei-Hsun Wang, Wen-Shin Hsu

**Affiliations:** 1 Department of Orthopedic Surgery, Changhua Christian Hospital, Changhua 500209, Taiwan; cmch10011@gmail.com; 2 Department of Golden-Ager Industry Management, Chaoyang University of Technology, Taichung 413310, Taiwan; 3 Department of Post-Baccalaureate Medicine, College of Medicine, National Chung Hsing University, Taichung 402202, Taiwan; 4 Department of Medical Information, Chung Shan Medical University, Taichung 402201, Taiwan; 5 Informatics Office Technology, Chung Shan Medical University Hospital, Taichung 402201, Taiwan

**Keywords:** wearable IoT care system, artificial intelligence, healthcare, long-term care environment, edge computing

## Abstract

With the rapid advancement of information and communication technology (ICT), big data, and artificial intelligence (AI), intelligent healthcare systems have emerged, including the integration of healthcare systems with capital, the introduction of healthcare systems into long-term care institutions, and the integration of measurement data for care or exposure. These systems provide comprehensive communication and home exposure reports and enable the involvement of rehabilitation specialists and other experts. Silver technology enables the realization of health management in long-term care services, workplace care, and health applications, facilitating disease prevention and control, improving disease management, reducing home isolation, alleviating family burden in terms of nursing, and promoting health and disease control. Research and development efforts in forward-looking cross-domain precision health technology, system construction, testing, and integration are carried out. This integrated project consists of two main components. The Integrated Intelligent Long-Term Care Service Management System focuses on building a personalized care service system for the elderly, encompassing health, nutrition, diet, and health education aspects. The Wearable Internet of Things Care System primarily supports the development of portable physiological signal detection devices and electronic fences.

## 1. Introduction

The Comprehensive Care Service System refers to a healthcare service that aims to provide integrated and coordinated care to patients through the use of various technologies and services. This system includes features such as electronic health records, telemedicine, remote patient monitoring, medication management, and caregiver support. The system also utilizes big data and artificial intelligence to improve clinical decision making, enhance patient safety, and optimize resource allocation. The advantages of the Comprehensive Care Service System are numerous. The system allows for real-time monitoring of patient health, which enables earlier detection of health problems and timely intervention. The use of electronic health records and other digital technologies also improves communication and collaboration between healthcare professionals, leading to better care coordination and more accurate diagnoses. The system also helps to reduce healthcare costs by preventing hospital readmissions, decreasing emergency room visits, and minimizing unnecessary testing and treatments. Patients can receive care in the comfort of their own homes, which increases their independence and improves their quality of life. Additionally, the system provides support to caregivers, which can help alleviate caregiver burden and improve patient outcomes.

A healthcare system that utilizes advanced technology and data management to provide more comprehensive and effective care services for patients is referred to as a healthcare system. It can include various components, such as electronic medical record systems, health monitoring devices, remote monitoring services, virtual medical consultations, etc. These technologies can help medical professionals better understand the health status of patients and provide more timely medical intervention and treatment plans. In terms of elderly care services, a healthcare system can help seniors better manage and monitor their health status. For instance, seniors can wear smart wristbands or watches to monitor their heart rate, blood pressure, and other physiological indicators. These data can be wirelessly transmitted to electronic medical record systems in medical institutions, where healthcare professionals can view them in real time and provide appropriate advice and treatment plans as needed. Additionally, remote monitoring services are also an essential part of elderly care services. Remote monitoring services can allow seniors to communicate and interact with healthcare professionals through video calls, text messages, or emails. This can help seniors better understand their illness and treatment plans and receive timely help and support when needed.

In summary, a healthcare system can bring more convenience and benefits to elderly care services. By utilizing these technologies and tools, seniors can better manage and monitor their health status, and healthcare professionals can provide timely treatment and support, thereby improving the quality of life and health status of seniors.

The main purpose of this study is to develop a comprehensive health and safety care system, based on precision medicine; to tailor a personalized health care model, which can prevent, manage, or monitor diseases, improve the health status of patients, reduce home care expenses, and promote health and chronic disease control; and to set up a comprehensive and safe innovative application service system. A multifunctional physiological measuring device worn by an elderly person can instantly grasp their health status. The device worn by an elderly person can also be positioned, play the role of an electronic fence, prevent an elderly person from getting lost, establish emergency functions, and provide safety protection measures. Mainly based on the principles of health and safety and action safety, it combines applications with intelligent devices to provide comprehensive health and safety care services for the elderly.

While long-term care facilities have increasingly adopted information technology in recent years, many have not fully utilized its potential. There are still many challenges to overcome, including a lack of standardization, insufficient IT infrastructure, and insufficient training for staff. In addition, many older adults may be resistant to using new technology, which can limit its effectiveness. As a result, long-term care facilities still heavily rely on traditional methods of care, which can be time-consuming and costly. To fully realize the benefits of information technology in long-term care, more efforts are needed to address these challenges and promote widespread adoption.

Our study proposes a comprehensive care service that allows wearable IoT care systems and the integrated intelligent long-term care service management system to interact and provide elderly individuals with a range of health care services, including nutritional intake, health education courses, medication management, and daily activity tracking. The elderly can maintain a continuous connection with the system, and, if there is any abnormal physiological monitoring signal, or if the elderly person exits the set range of the electronic fence, the integrated smart long-term care service management system immediately issues alert messages to the elderly person and authorized individuals, thereby achieving an emergency response and reminder mechanism.

Long-term care facilities have increasingly turned to information technology to improve healthcare services. Some of the reasons for this trend include the need to efficiently manage large volumes of patient data, reduce medical errors, improve care coordination among healthcare providers, and provide patients with more personalized care. Additionally, technology such as electronic health records (EHRs), telehealth, and mobile health (mHealth) can help to improve patient outcomes, increase patient engagement, and reduce healthcare costs. Overall, the use of information technology in healthcare has the potential to transform the way that long-term care facilities deliver services, improving both the quality of care and patient satisfaction [1,2,3,4,5,6,7,8,9,10].

The wearable Internet of Things (IoT) care system is an innovative and versatile application service system that can be worn on the body to monitor and manage personal health. The system utilizes IoT technology, allowing multiple sensors and devices to connect and communicate with each other, enabling a variety of functions and applications [11,12,13,14,15,16].

- Wearable Device

The wearable IoT care system can monitor personal physiological indicators, such as heart rate, blood pressure, and body temperature. The system can also monitor personal physical activity and record dietary habits and medication information. All of these data can be wirelessly transmitted to a central monitoring station for analysis and processing to help caregivers better understand personal health status. In addition, the wearable IoT care system also provides real-time communication and location functions for personal and caregivers to communicate and locate. The system supports multiple communication methods, such as voice, text, and video, for different needs and scenarios. At the same time, the system can locate personal positions through technologies such as GPS to better respond to and manage personal needs. The wearable IoT care system is an innovative application service system that is both versatile and secure. It can help caregivers better monitor and manage personal health, while providing real-time communication and location functions to better manage and care for individuals. The system is highly reliable and secure, ensuring personal privacy and data security.

Recent advancements in low-power electronics and the miniaturization of sensors have enabled the development of a diverse range of wearable devices for health and fitness monitoring applications. Extensive research and commercial efforts worldwide, particularly in North America, are focused on creating wearable biosensors that can continuously monitor biochemical markers in the human body to aid in disease diagnosis, prognosis, and fitness tracking. Min et al. [17] conducted a review on wearable electrochemical biosensors. The review discussed the current progress and commercialization of wearable electrochemical sensors for measuring blood glucose levels as well as monitoring dehydration in fitness applications. Although the authors found promising potential for wearable electrochemical sensors in remote and personalized healthcare, they did not cover recent advancements and the future of care service management system.

Gao et al. [18] conducted a review of the current developments in skin-interfaced wearable sensors. They discussed the selection of materials and design strategies that enable sensors to be in contact with human skin and categorized and analyzed physical and biochemical sensors. The paper also delved into power issues, wireless communication, and data analysis. The authors identified challenges and opportunities for future wearable devices and systems. However, the paper lacked a complete framework for developing a care service management system.

Researchers conducted numerous studies and analyses on wearable devices in various fields, including healthcare, sports, and entertainment. Some studies focused on the technical aspects of wearable devices, such as their accuracy and reliability, while others examined the potential applications of wearable devices in different contexts. In healthcare, wearable devices were studied for their ability to monitor vital signs, detect diseases, and manage chronic conditions. Researchers explored the use of wearable devices in various clinical settings, including hospitals, nursing homes, and home care settings. They also investigated the potential of wearable devices to improve patient outcomes, reduce healthcare costs, and enhance the overall quality of care. In sports and fitness, wearable devices were studied for their ability to track physical activity, monitor performance, and prevent injuries. Researchers examined the accuracy of wearable devices in measuring various metrics, such as heart rate, calories burned, and distance traveled. They also investigated the potential of wearable devices to motivate individuals to adopt and maintain healthy behaviors. In entertainment, wearable devices were studied for their ability to enhance the user experience in various contexts, such as gaming and virtual reality. Researchers explored the use of wearable devices to provide haptic feedback, track body movements, and create immersive experiences. They also investigated the potential of wearable devices to improve accessibility for individuals with disabilities. Overall, the literature on wearable devices suggested that these technologies have great potential to revolutionize various industries and improve people’s lives. However, more research is needed to address technical, ethical, and regulatory issues and to fully understand the impact of wearable devices on individuals and society [17,18,19,20,21].

- Edge Computing

Edge computing refers to the practice of processing data near the edge of the network, closer to where the data are generated, rather than in a centralized data center. This allows for faster data processing and reduced latency, as well as the ability to locally filter and analyze data before they are sent to a central data center for further processing or storage. Edge computing is becoming increasingly important with the growth of the Internet of Things (IoT), which involves a large number of connected devices generating vast amounts of data. By processing data at the edge, devices can operate more efficiently and with lower latency, which is particularly important in applications such as autonomous vehicles and industrial automation. Edge computing can be implemented through a variety of technologies, such as micro data centers, cloudlets, and fog computing. These technologies enable computation, storage, and networking capabilities to be deployed closer to the devices generating the data [22,23,24].

In recent years, the concept of edge computing has captured the attention of researchers as a viable alternative to conventional cloud-based systems, with the aim of minimizing interaction time and collecting large amounts of real-time data from IoT devices. Edge-based approaches have the potential to support time-sensitive applications in the Industry 4.0 landscape in the coming years [25]. The use of fog and mobile edge computing has become increasingly important in addressing challenges associated with cloud computing, including response time, mobility, and location perception. In the healthcare domain, an IoT-based system and decision-making model were proposed for the detection and monitoring of type 2 diabetes patients. The model utilized a hybrid approach combining type 2 neutrosophic and Višekriterijumsko kompromisno rangiranje (VIKOR) methods. The results of the study demonstrated the effectiveness and robustness of the proposed framework, with a 9.8% reduction in execution time and increased patient survival rates based on personal data and symptoms [26]. However, the authors suggested the need to explore more advanced machine learning methods to improve prediction accuracy. Yang et al. [25] introduced an emotion-aware system that includes a personal robot, smart clothing, and edge cloud for interacting with users. The system collects EEG data, audio/video data, touch data, and physiological signals from the user through smart clothing. The authors designed emotion perception and interaction algorithms using artificial intelligence and a knowledge graph, such as intelligent recommendation, relation recognition, and emotional expression recognition. To evaluate the system’s performance, a testbed was developed, and various tests were conducted. The results showed that the proposed emotion-aware system is effective in improving people’s mental health. Chen et al. [22] proposed an ECC-based smart healthcare system that utilizes edge cognitive computing and a data-driven approach. The system monitors and analyzes physical health-related data from users using cognitive computing. The experimental results showed that the proposed system has superior user data cognition and resource cognition, high energy efficiency, low cost, high user Quality of Experience (QoE) in emergency cases, and reasonably improved survival rates. However, the authors suggested that an emotional recognition system could be built by combining this system with existing technology to provide corresponding care.

- Electronic Fences

Electronic fences can be used for monitoring and managing elderly patients. For example, for elderly patients with dementia or cognitive impairment, setting up an electronic fence can prevent them from getting lost or wandering, while providing real-time location monitoring and alarms for timely intervention and management. In addition, for chronic disease patients who need long-term monitoring and care, such as cardiovascular disease patients and diabetes patients, an electronic fence can be set up to monitor their activities and behaviors and to identify and address disease conditions in a timely manner.

In the field of long-term care services, electronic fences can be used for monitoring and managing elderly individuals with dementia or cognitive impairment. By setting up an electronic fence, caregivers can prevent elderly individuals from wandering outside of a designated safe area, while providing real-time location monitoring and alarms for timely intervention and management. This technology can help reduce the risk of injury or harm to an elderly individual, while also providing peace of mind for family members and caregivers.

In addition, electronic fences can also be used to monitor the activity range and behavior of individuals in long-term care facilities or homes. Caregivers can set up electronic fences to ensure that individuals do not leave the designated area, while also providing alerts if individuals engage in unusual or potentially harmful behavior. This technology can help improve the safety and quality of care for individuals in long-term care settings. Electronic fences are a valuable technology for improving the safety and quality of long-term care services. By utilizing this technology, caregivers can better monitor and manage elderly individuals with dementia or cognitive impairment, while also providing a safer and more secure environment for individuals in long-term care facilities or homes [12,25,26,27,28,29,30,31,32,33].

## 2. Materials and Methods

**Figure 1:**
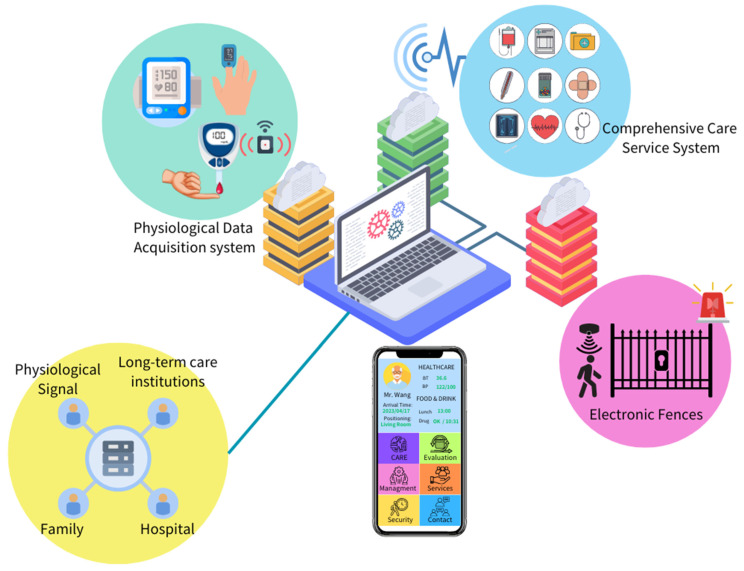
The integrated intelligent long-term care service management system.

This study aims to develop a comprehensive health and safety care system based on precision medicine, tailored to individualized health care models. It can achieve prevention, management, or monitoring of diseases, improve patients’ health status, reduce home care (nursing) expenses, and promote health and chronic disease control. By using a wearable IoT care system and an integrated smart long-term care service management system, the elderly can receive comprehensive health care services, including nutrition intake, health education courses, medication management, daily activity monitoring, and more. The elderly stay connected with the system at all times. Once the physiological monitoring signal of an elderly person shows abnormalities, or the electronic fence is out of the preset range, the integrated smart long-term care service management system immediately sends alert messages to the elderly and relevant authorized personnel, achieving an emergency response and reminder mechanism, as shown in Figure 1.

The wearable IoT care system is responsible for monitoring the daily status of the elderly by providing basic physiological data, including blood pressure, pulse, body temperature, and emotional response monitoring as well as heart function assessment and atherosclerosis and other chronic disease monitoring using the wearable devices that the elderly use. The back-end system can use these data to find the best medical care service for the elderly. An elderly person’s family members can also view their physiological data and daily activities through the Internet and the entrance service station. Through the integrated intelligent long-term care service management system, the family members of an elderly person can be authorized to access some information about the elderly person within the long-term care institution, including nutrition intake, health education courses, and daily activity status. an elderly person’s nutritional intake and water consumption in the long-term care institution are closely related to physiological data monitoring, and a personalized diet plan and nutrition intake record can be tailored to help the elderly person achieve a healthy and safe living pattern within the long-term care institution.

Regarding the medical and nutrition aspects, in addition to relying on information technology applications, it is still necessary to seek the assistance of professional medical and nursing staff to provide professional knowledge. Therefore, the integrated intelligent long-term care service management system is planned to provide long-term care institutions with comprehensive health care services for the elderly, including nutrition intake, health education courses, medication management, and daily activity monitoring.

The smart wearable device used in this study provides a variety of functions. Using Bluetooth sensing technology and a secure radar, it can remotely trace the trajectory of an elderly person and set up an electronic fence for notification. With location sensors, the device can provide elderly people with a sense of security by enabling them to be tracked at any time and place. In addition, the device can monitor an elderly person’s mobility and basic physiological information and transmit these data to the backend database in real time, allowing the center staff to receive immediate updates on the elderly person’s status. The system is primarily built on the Internet of Things (IoT) architecture, which combines the Internet and internal network to create a safe, healthy, and high-quality care environment for the day care center.

The integrated intelligent long-term care service management system applies artificial intelligence technology to assess and monitor the physical and mental status of the elderly and stores their physiological information in the care data management system to establish a digital electronic contact book for the elderly. With the assistance of professional physicians, the system can achieve health promotion and chronic disease management. Through the integrated intelligent long-term care service management system, the physical and mental status of the elderly can be monitored and assessed in real time. The system can automatically identify potential health risks and provide corresponding recommendations. The physiological and care data of the elderly can be stored in the system, making it convenient for medical staff and family members to view and provide effective care and management. In addition, the integrated intelligent long-term care service management system can also be used with professional physicians for health management and chronic disease control. Physicians can develop personalized health management plans based on the elderly person’s condition and care data and provide remote diagnosis and treatment. The system also provides real-time communication, making it easy for the elderly person and their family members to contact medical staff to promptly solve problems.

**Figure 2:**
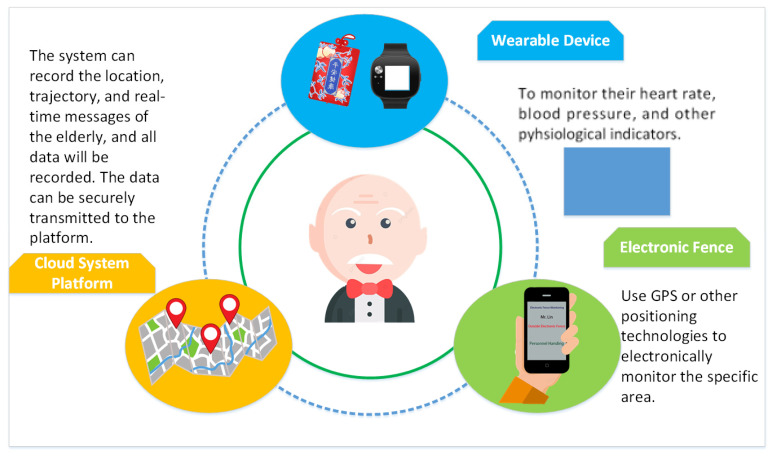
The wearable IoT care system.

As shown in Figure 2, the following methods are adopted.

1. Facial recognition and RFID card check-in: In addition to using facial recognition technology, the system utilizes RFID cards for check-in purposes.
2. Wearable device for data input: A wearable device is employed to input measurement results into the computer system. These data are then confirmed, combined with personal information, and used for check-in.
3. Customized design for user preferences: The design of the wearable device varies based on the preferences of the user. For instance, options such as badges, pendants, belts, or bracelets are provided to cater to individual preferences and avoid making the elderly feel monitored or restricted.
4. RFID system for positioning and tracking: The RFID system is utilized for accurate positioning and tracking of elderly individuals, both indoors and outdoors. Through area recognition and tracking methods, the system ensures their correct location is identified. If a person remains in a stagnant state for over 10 min, the system alerts caregivers for necessary checks or care based on their location.
5. Backend server for data computation: The collected data, including the whereabouts of elderly individuals, are sent to the backend server for computation. Artificial intelligence, big data analysis, and deep learning techniques are employed to utilize these data as a reference model, thereby enhancing the quality of elderly care services.

It is important to note that these methods are implemented to provide comprehensive care services. The aim is to ensure that the elderly experience a sense of health, safety, and respect while using information technology.

## 3. Results

This study develops an intelligent wearable physiological signal measurement integrated system that integrates multiple functional physiological signal measurement technologies into a device, including detection of heart rate, body temperature, blood oxygen, and blood pressure. It also combines wireless transmission technology to transmit physiological signals and location information back to the monitoring center in real time. Additionally, in response to the needs of long-term care or day care facilities, due to various activities, some areas being more dangerous that need to be avoided by care recipients alone, and the fact that care recipients may also leave the park area, the system also includes location tracking and electronic fence functions. Finally, a database system is established to collect and establish various physiological information as a data source for subsequent research analysis.

Since each elderly person has a different age and different physical and mental conditions, improper use may cause the body to be unable to bear a device, which may lead to shock. Elderly people may also wander or have accidents while walking or in the bathroom. Therefore, this project aims to reduce accidents and, in the event of an accident, to detect and call for emergency assistance as quickly as possible through an intelligent wearable device, enabling elderly people to have a safe and healthy life.

As shown in Table 1, the “RFID Positioning and Tracking Data” column represents the location where the person is detected by the RFID system, and the “Electronic Fence Monitoring” column indicates whether the person is within the designated area. The “Personnel Handling” column can be used to indicate any necessary actions or interventions by caregivers or staff.

The elderly-related information in this study is recorded, and suitable diets and meals are arranged based on individual health conditions. The structured storage and management through the system can save the amount of time spent on elderly care in the future. Moreover, with the advancement of medical technology, there are many chronic diseases such as hypertension and diabetes that cannot be reversed and can only be controlled through nutrition and healthy lifestyle habits. Currently, nutritionists can only calculate the daily energy and nutrient requirements and allocate them for the elderly or patients to refer to. However, this information is insufficient for the elderly and some patients. Therefore, this study uses artificial intelligence technology, combined with nutrition experts and food nutrition databases, to establish a comprehensive nutrition knowledge base. This can significantly reduce the waste of medical resources for chronic disease patients.

One of the primary advantages of this system is its ability to collect and store a vast amount of physiological data from multiple sources in one location. This allows healthcare professionals to more efficiently and effectively analyze patient data, enabling them to make better-informed decisions about patient care. The system is also highly user-friendly, making it accessible to a wide range of patients, including the elderly, who may not be so technologically savvy. The Smart Wearable Physiological Signal Measurement Integration System has a high practical value and can be applied in various healthcare settings, including hospitals, nursing homes, and home care settings. In hospitals, the system can provide continuous monitoring of patients’ vital signs, allowing healthcare professionals to detect early signs of deterioration and promptly respond to prevent adverse events. In nursing homes and home care settings, the system can improve patient safety by alerting caregivers when a patient leaves a designated area or when vital signs indicate an emergency.

**Figure 3:**
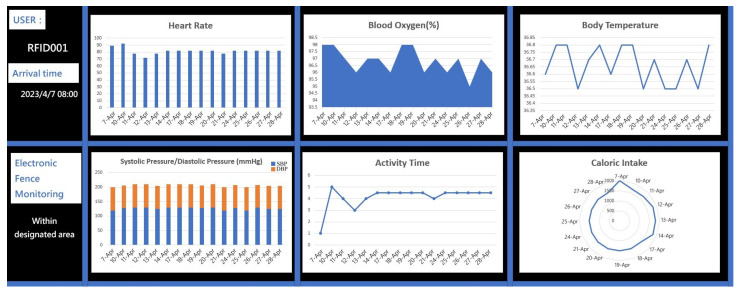
Data dashboard.

As shown in Figure 3, the data from all IoT devices are recorded and analyzed using a data dashboard. The dashboard provides a comprehensive view of the collected data and allows for in-depth analysis and insights. The recorded data include various parameters such as heart rate, body temperature, blood oxygen level, systolic pressure, diastolic pressure, step counts, activity time, electronic fence monitoring, and caloric intake. The data dashboard presents this information in a visually appealing and easy-to-understand format, such as charts, graphs, and tables. It allows users to track trends, identify patterns, and monitor changes over time. By leveraging the power of data analytics, the dashboard provides valuable insights into the health and well-being of individuals. It enables healthcare professionals and caregivers to make informed decisions and take proactive measures based on the analysis of the collected data. Furthermore, the dashboard can generate alerts and notifications based on predefined thresholds or abnormal readings, ensuring timely interventions when necessary. Overall, the data dashboard plays a crucial role in managing and analyzing the data from IoT devices, providing valuable information for healthcare professionals, caregivers, and individuals themselves to improve health outcomes and enhance the quality of care.

Long-term care facilities have been using information technology in health care for several reasons, including the following.

1. Addressing the challenges of an aging society with a declining birthrate: An aging society with a declining birthrate faces a shortage of long-term care resources, and using information technology can increase the efficiency of utilizing care resources.
2. Improving the quality of care: Using information technology can collect and analyze the health status of the elderly, understand their care needs in a timely manner, and provide more accurate and personalized care services.
3. Enhancing safety and security: Using information technology can improve the quality of care, reduce the occurrence of accidents, and enhance home safety and security.
4. Shortening the distance between caregivers and the elderly: Using information technology can shorten the distance between caregivers and the elderly, increase opportunities for interaction, and reduce the sense of loneliness among the elderly.
5. Reducing care costs: Using information technology can improve care efficiency, reduce care costs, and comply with long-term care policy requirements.

## 4. Discussion

The integration of artificial intelligence (AI) and wearable Internet of Things (IoT) systems in long-term care environments holds significant potential for improving the quality of care and enhancing the well-being of individuals. In this study, we explored the possibilities and benefits of combining AI and wearable IoT technologies in long-term care settings. One of the key advantages of integrating AI and wearable IoT systems is the ability to collect and analyze a wealth of physiological data in real time. These data include parameters such as heart rate, body temperature, blood oxygen level, and blood pressure, among others. By continuously monitoring these vital signs, healthcare professionals can gain valuable insights into the health status of individuals and detect any abnormalities or changes that may require attention. This enables early intervention and proactive care management, which can ultimately lead to better health outcomes.

The use of AI algorithms in analyzing the collected data further enhances the capabilities of the wearable IoT system. AI algorithms can identify patterns, trends, and anomalies in the data, providing valuable predictive and diagnostic capabilities. For example, AI algorithms can detect early signs of health deterioration or predict the risk of certain health conditions based on the collected data. This allows for personalized and targeted interventions, optimizing the delivery of care and support. Moreover, the integration of AI and wearable IoT systems enables remote monitoring and communication, facilitating seamless interaction among individuals, caregivers, and healthcare professionals. Through the use of mobile applications or web portals, individuals can easily share their health data with their care team, receive personalized recommendations, and communicate any concerns or questions. This remote monitoring capability is particularly beneficial in long-term care environments where individuals may require ongoing support and supervision.

Furthermore, there may be technical challenges in ensuring the accuracy and reliability of wearable IoT devices. The calibration and validation of these devices are essential to ensure accurate data collection. Ongoing maintenance and monitoring of these devices are also necessary to detect any malfunctions or deviations that may affect the data quality.

The integration of AI and wearable IoT systems in long-term care environments offers significant opportunities for enhancing care delivery, promoting proactive health management, and improving the overall well-being of individuals. By leveraging the power of AI algorithms and real-time data monitoring, healthcare professionals can provide personalized and timely interventions, ultimately leading to better health outcomes and a higher quality of care in long-term care settings. However, it is crucial to address privacy concerns, ensure data accuracy, and address technical challenges to fully realize the potential of this integration in long-term care environments.

## 5. Conclusions

Through the utilization of artificial intelligence technology, this system establishes a comprehensive nutrition knowledge base, which enables elderly individuals to receive personalized recommendations for their dietary habits and nutrient intake, based on their personal health status, age, gender, weight, medical history, and other relevant information. The system allows an elderly person and their family to search for the nutritional composition of different foods, learn about the daily nutrient intake of the elderly person, and gain a detailed understanding of the elderly person’s dietary plan. Moreover, the system can provide recommendations and adjustments based on the elderly person’s nutrient intake, helping them better control their dietary habits and improve their overall health status. By employing artificial intelligence technology and establishing a comprehensive nutrition knowledge base, this system delivers accurate and personalized nutrition recommendations and facilitates health management for the elderly through machine learning.

The development of the smart wearable physiological signal measurement and integration system represents a promising solution for the healthcare industry. By integrating multiple physiological signal measurement technologies into a single wearable device and combining it with wireless transmission and location-based services, this system offers a comprehensive and real-time monitoring solution for patients or elderly individuals. Furthermore, the collected data can be stored in a database for further analysis and research purposes. Through the integration of IoT and big data technologies, this system can provide valuable insights and enhance the overall efficiency and quality of healthcare services. Overall, the smart wearable physiological signal measurement and integration system serves as a practical and valuable tool for healthcare units and hospitals, enabling them to enhance patient care and safety.

By integrating IoT and big data technologies, valuable data can be generated by connecting various smart devices through IoT technology. Through the analysis of big data platforms, this system can provide the most complete, real-time service channels and demand information. The introduction of smart technology not only improves the efficiency of the original manual processes but also enables service management through an information platform. Moreover, data analysis can enhance service quality and even lead to the development of innovative services.

With the development and advancement of information technology, the wearable IoT care system and the integrated intelligent long-term care service management system can interactively provide comprehensive healthcare services to elderly individuals. These services include nutrition intake, health education courses, medication management, daily activity monitoring, etc. This ensures that the lives of elderly individuals are safer and more convenient. The wearable IoT care system can track the activity trajectory of elderly people at all times, promptly notifying caregivers in the event of abnormalities or falls, who can take immediate action and reduce the probability of accidents. Caregivers can monitor an elderly person’s physiological data, such as weight, heart rate, and breathing, in real time through the system, enabling them to take appropriate actions when necessary. By applying information technology, long-term care institutions can provide more comprehensive care services, enabling elderly individuals to achieve a healthy and safe living environment within these institutions.

## Figures and Tables

**Table 1:** RFID positioning and tracking data.

Timestamp	RFID ID	Heart Rate	Activity Time	RFID Positioning and Trancking Data	Electronic Fence Monitoring	Personnel Handing
17-Apr-23	RFID001	88 bpm	1.5 h	Living Room	Inside Electronic Fence	None
17-Apr-23	RFID002	92 bpm	1.5 h	Class Room	Inside Electronic Fence	None
17-Apr-23	RFID003	78 bpm	2 h	GYM	Inside Electronic Fence	None
17-Apr-23	RFID004	92 bpm	1.5 h	Class Room	Inside Electronic Fence	None
17-Apr-23	RFID005	82 bpm	1 h	Living Room	Inside Electronic Fence	None
17-Apr-23	RFID006	88 bpm	0.5 h	Garden	Inside Electronic Fence	None
17-Apr-23	RFID007	75 bpm	1 h	Living Room	Inside Electronic Fence	None
17-Apr-23	RFID008	80 bpm	1 h	GYM	Inside Electronic Fence	None
17-Apr-23	RFID009	92 bpm	1.5 h	Living Room	Inside Electronic Fence	None
17-Apr-23	RFID010	78 bpm	1 h	Class Room	Inside Electronic Fence	None
17-Apr-23	RFID011	85 bpm	0.5 h	GYM	Inside Electronic Fence	None
17-Apr-23	RFID012	75 bpm	1 h	Class Room	Inside Electronic Fence	None
17-Apr-23	RFID013	90 bpm	1 h	Living Room	Inside Electronic Fence	None
17-Apr-23	RFID014	78 bpm	0.5 h	Garden	Inside Electronic Fence	None

## Data Availability

Not applicable.
